# Monoamines, Insulin and the Roles They Play in Associative Learning in Pond Snails

**DOI:** 10.3389/fnbeh.2019.00065

**Published:** 2019-04-02

**Authors:** Yuki Totani, Hitoshi Aonuma, Akira Oike, Takayuki Watanabe, Dai Hatakeyama, Manabu Sakakibara, Ken Lukowiak, Etsuro Ito

**Affiliations:** ^1^Department of Biology, Waseda University, Tokyo, Japan; ^2^Research Institute for Electronic Science, Hokkaido University, Sapporo, Japan; ^3^CREST, Japan Science and Technology Agency, Kawaguchi, Japan; ^4^Department of Biological Sciences, Faculty of Science, Hokkaido University, Sapporo, Japan; ^5^Faculty of Pharmaceutical Sciences, Tokushima Bunri University, Tokushima, Japan; ^6^Research Organization for Nano and Life Innovation, Waseda University, Tokyo, Japan; ^7^Hotchkiss Brain Institute, University of Calgary, Calgary, AB, Canada; ^8^Graduate Institute of Medicine, School of Medicine, Kaohsiung Medical University, Kaohsiung, Taiwan

**Keywords:** 5-HT, conditioned taste aversion, dopamine, insulin, *Lymnaea*, octopamine

## Abstract

Molluscan gastropods have long been used for studying the cellular and molecular mechanisms underlying learning and memory. One such gastropod, the pond snail *Lymnaea stagnalis*, exhibits long-term memory (LTM) following both classical and operant conditioning. Using *Lymnaea*, we have successfully elucidated cellular mechanisms of learning and memory utilizing an aversive classical conditioning procedure, conditioned taste aversion (CTA). Here, we present the behavioral changes following CTA training and show that the memory score depends on the duration of food deprivation. Then, we describe the relationship between the memory scores and the monoamine contents of the central nervous system (CNS). A comparison of learning capability in two different strains of *Lymnaea*, as well as the filial 1 (F_1_) cross from the two strains, presents how the memory scores are correlated in these populations with monoamine contents. Overall, when the memory scores are better, the monoamine contents of the CNS are lower. We also found that as the insulin content of the CNS decreases so does the monoamine contents which are correlated with higher memory scores. The present review deepens the relationship between monoamine and insulin contents with the memory score.

## Introduction

Molluscan gastropods are generally referred to as snails or slugs. The words “snail” and “slug” are often deemed pejorative (i.e., depreciatory). Three examples are: a construction job makes progress at a *snail’s pace*. In Japan, the use of *snail mail* is still required for job hunting. Stock prices are *sluggish*. However, in spite of the negative connotations associated with snail and slug, these animals significantly contributed to the birth of neuroscience in regards to basic functioning of neurons that were identifiable, their ability to control interesting and tractable behaviors, and lastly to an understanding of how changes in neuronal function and connectivity underlie learning and memory (see Willows, 1973; Clarac and Pearlstein, 2007 as the review; Bruner and Tauc, 1964; Kandel and Tauc, 1965 for the sea hare *Aplysia*, Gelperin, 1975 for the terrestrial slug *Limax*, Alkon, 1976 for the sea slug *Hermissenda*, Balaban, 2002 for the snail *Helix*, Gillette and Brown, 2015 for the sea slug *Pleurobranchaea*).

The pond snail *Lymnaea stagnalis* has been utilized for studies of learning and memory for almost four decades (see Alexander et al., 1982; Audesirk et al., 1982 for early studies; see Benjamin et al., 2000 for recent studies). Landmark studies (Kater and Rowell, 1973; Benjamin and Rose, 1979; Rose and Benjamin, 1979) elucidating the neural control of feeding in two pulmonate mollusks (*Lymnaea* and *Helisoma*) ultimately lead to an understanding of the interconnectivity of identified neurons that constituted a central pattern generator (CPG) that drove feeding behaviors (Delcomyn, 1980; Murphy, 2001; Benjamin et al., 2008). In *Lymnaea*, there was also the elucidation of a second CPG, which drove aerial respiration (Syed et al., 1990, 1992; Lukowiak et al., 1996, 2014; Sunada et al., 2017b). The behaviors mediated by the two aforementioned CPGs have also been shown to have age-depended differences in their ability to learn and form memory making them ideal candidates for the study of age-dependent alterations in cognition (Yamanaka et al., 1999; McComb et al., 2005; de Weerd et al., 2017).

The elucidation of the neural network underlying feeding behavior in *Lymnaea* enabled both the Ito and Sakakibara groups in Japan to examine the causal neural mechanisms of a taste aversive conditioning (Kojima et al., 1996; Kawai et al., 2004). The Ito laboratory succeeded in constructing a training procedure for conditioned taste aversion (CTA) learning and long-term memory (LTM) formation in *Lymnaea* (Ito et al., 1999). The feeding behavior of snails is innately activated by sucrose, whereas feeding is inhibited by aversive stimuli (e.g., a KCl solution or electric shock) that elicit the whole-body withdrawal response. Its activation not only inhibits feeding but also suppresses heart rate (Kita et al., 2011). In the CTA procedure, there is temporal contiguity between the presentation of the sucrose stimulus (conditioned stimulus: CS) followed by the presentation of the KCl or electric shock stimulus (unconditioned stimulus: US). Following this pairing, the feeding response in snails to sucrose (CS) is significantly reduced (Ito et al., 2013), and the LTM is maintained for more than 1 month (Kojima et al., 1996). The changes in neuronal activity that underlie the behavioral changes have also been elucidated (Kojima et al., 1997, 1998, 2000, 2001, 2015; Ito et al., 2012; Otsuka et al., 2013). Independently, the Sakakibara group started working CTA in *Lymnaea* using sucrose as the CS and a tactile stimulus or an electric shock as the US (Kawai et al., 2004). They also clarified the issue of spaced learning vs. massed learning in CTA (Takigami et al., 2014).

In the present review, we cover the following four points: (1) the strength or score of CTA memory is dependent on the duration of food deprivation; (2) there is a negative correlation between the memory scores and the monoamine contents in the central nervous system (CNS); (3) when the memory scores in three distinct *Lymnaea* populations (i.e., Dutch and Canadian strains, and their F_1_ cross snails) are compared, a negative correlation between memory scores and monoamine contents continues to be observed; and (4) insulin in the CNS improves the memory score in memory-impaired snails. We conclude that when the memory score following CTA training is better, certain monoamine contents are lower in the CNS of the snail. Finally, we suggest a correlation between insulin, monoamine contents and the memory scores. Insulin decreases serotonin (5-hydroxytryptamine: 5-HT) content, and thus the memory score is enhanced with insulin.

## CTA Learning in Snails

### CTA Training Protocol

The initial CTA training procedure was carried out “manually.” That is, an investigator applied to the snail using a pipette the paired stimuli, which sometimes caused increased variability to occur among data obtained by different snail trainers (Kojima et al., 1996; Wagatsuma et al., 2004; Sugai et al., 2006). Recently, however, the pairing is accomplished with an automated learning apparatus, which tends to alleviate experimental differences among investigators (Takigami et al., 2016). Here, we present the data obtained using both methodologies. Whereas there may have been more individual variability in earlier studies, there are no significant differences in conclusions obtained between these two methodologies.

**Figure 1:**
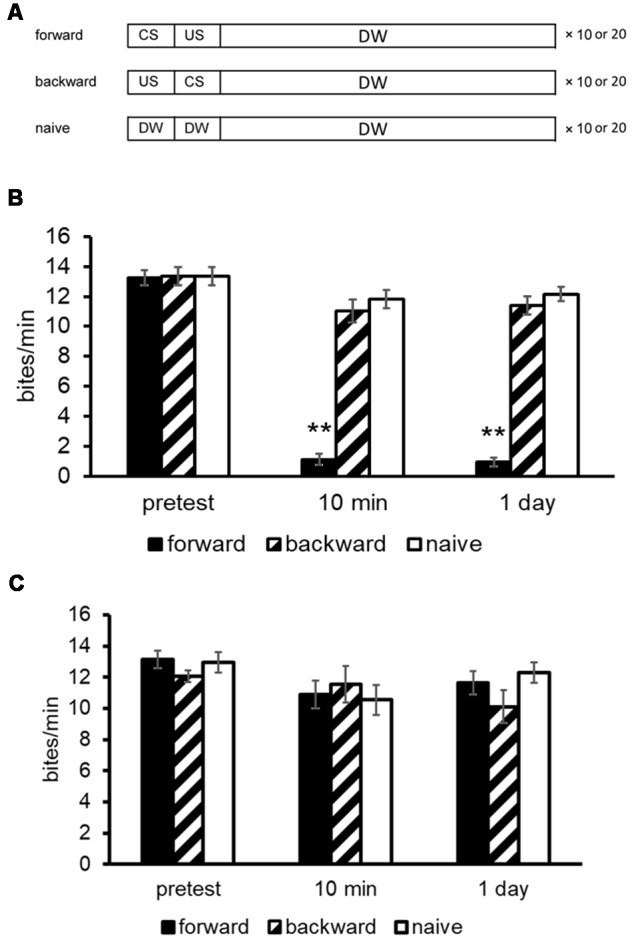
Conditioned taste aversion (CTA) training procedures for *Lymnaea*. **(A)** The conditioned stimulus (CS) was a 10 mM sucrose solution and the unconditioned stimulus (US) was a 10 mM KCl or a 3-s high voltage electric shock. The interstimulus interval between the onset of CS and that of US was 15 s; and the intertrial interval was 10 min. Snails received 10 paired CS-US trials (i.e., forward) on a single day. The CS was applied to the lips for 15 s, and the feeding response was determined for 1 min as a pretest or a post-test. A backward-conditioned (US-CS) cohort and a naive (presented only with distilled water) control cohort were also performed. **(B)** The memory scores of CTA (i.e., number of bites to the CS) in Day 1 snails were examined at a pre- and post-tests. Black bars showed CTA-trained snails; hatched bars showed backward-conditioned snails; and white bars showed naive control snails. The number of snails was at least 10 in each condition. The data are expressed as mean ± SEM. The time indicated on the *x*-axis represents the time of the post-test session that was performed following the training. **Indicates *P* < 0.01. **(C)** The memory scores of CTA in Day 5 snails were examined at a pre- and post-tests. CTA memory formation did not occur as no suppression of feeding responses was observed. This figure was modified from Ito et al. (2015b) and Aonuma et al. (2018a). The articles were published in *J. Exp. Biol*. and *Neurobiol. Learn. Mem*., respectively, and thus the reuse of contents is permitted by the Green Open Access model and the “author and user rights” of Elsevier, respectively.

A brief description of the automated learning apparatus, which can train multiple snails simultaneously, is given here (Takigami et al., 2016). The manual training used the different parameters that are indicated in the text and figures, if necessary. To deliver the CS (i.e., 100 mM sucrose in tap water) to the snails, the sucrose is propelled in tubes (50 ml conical tubes) by a water pump. The CS solution is delivered to the snails for 15 s. The US is a high voltage electric shock with 3 s duration. The interstimulus interval between the CS and US was 15 s, and the intertrial interval was 70 s. The CS elicits a feeding response reliably, whereas the US elicits a withdrawal response. Snails received 10 pairs of CS-US controlled with a microcomputer. Here, we should note that when the CTA training was performed manually, the number of CS-US pairings was 10 or 20 (Mita et al., 2014a; Ito et al., 2015b). Controls included a backward-conditioning (US-CS) group and a naive group to validate associative learning. For the naive control group, only distilled water was applied instead of the CS and US (Figure 1A; Kojima et al., 1996).

Before training, a 15-s application of the CS for the pretest evoked a similar number of feeding responses for its following 1 min (about 12–18 bites/min) in all snails. In the post-tests, snails were again challenged with the CS. The number of feeding responses (i.e., bites) was counted as a measurement of memory strength in the 1-min interval in distilled water after a 15-s application of the CS. All tests were performed blindly. After the CTA training (i.e., CS-US pairings), the feeding response elicited by the CS was significantly suppressed (Figure 1B; Aonuma et al., 2018a), and this suppression lasted for more than a month (i.e., LTM; Kojima et al., 1996). On the other hand, the feeding was not suppressed in the backward-conditioned group nor in the naive control group.

### Food-Deprivation State and CTA

Food deprivation before and during CTA training is of vital importance for learning and memory formation to occur (Sugai et al., 2007; Ito et al., 2017). We thus examined the relationship between the duration of food deprivation and the CTA memory score. For example, with 1 day of food deprivation before CTA training, snails acquired CTA learning and LTM persisted for at least 1 month. However, with 5 days of food deprivation before CTA training, snails did not learn or remember (Figure 1C; Ito et al., 2015b). We have called these snails that received the food deprivation for 1 day and 5 days Day 1 and Day 5 snails, respectively. In other words, Day 1 snails were mildly hungry and Day 5 snails were severely hungry. As will be discussed below, the state of food deprivation is correlated with levels of insulin [actually, molluskan insulin-related peptide (MIP)] in the CNS of the snail that is a key determining factor in CTA learning and memory formation (Mita et al., 2014a,b).

## Monoamines and CTA Learning

### Monoamines in Gastropods

**Figure 2:**
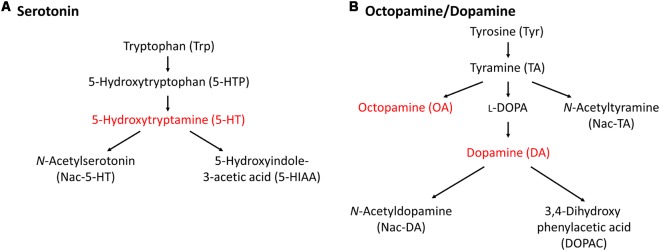
Metabolic pathways of monoamines. **(A)** 5-HT metabolic pathway. **(B)** Octopamine/dopamine metabolic pathway. Only the monoamines that we could measure in the high-performance liquid chromatography (HPLC) assay for the *Lymnaea* central nervous system (CNS) are indicated. This figure was modified from Aonuma et al. (2018b). This article was published in *Biophys. Physicobiol*., and thus the reuse of contents is permitted by the CC BY 4.0 license.

In this section, we describe the relationship between monoamine content and CTA memory scores in *Lymnaea*. Before that, we need to discuss the differences in monoamine catabolism between vertebrates and invertebrates (especially gastropods). This is described in Figure 2 (Aonuma et al., 2018b). In vertebrates, most monoamine neurotransmitters are metabolized by monoamine oxidase (MAO; Cooper et al., 2003). In contrast, there are alternative metabolic pathways to produce monoamines in invertebrates in addition to the MAO pathway (Sloley, 2004). Octopamine (OA), but little or no noradrenaline, is found in invertebrates, and thus it is speculated that OA is an “adrenergic” transmitter of invertebrates (Roeder, 1999). Interestingly, the monoamine catabolism in gastropods seems further different from cephalopods and bivalves (Sloley, 2004). For example, previous reports described that the MAO pathway does not exist or, if anything, plays a very minor role in *Aplysia* nervous tissues (McCaman and Dewhurst, 1971), and this role seems to be taken over by γ-glutamyl conjugation (McCaman et al., 1985; Sloley et al., 1990; Sloley and Goldberg, 1991).

### 5-HT and CTA

5-HT is assumed to be involved with CTA for the following reasons. 5-HT plays key roles in the mediation of learning and memory in mollusks (Kandel, 2001). 5-HT also likely plays an important role in memory reconsolidation in *Aplysia* as well as in other forms of neuronal plasticity (Cai et al., 2012; Hawkins, 2013). Specific reduction of 5-HT in the CNS of the terrestrial slug *Limax valentianus* impaired short-term memory (STM) but not LTM in an aversive odor-taste associative learning (Shirahata et al., 2006). In the terrestrial snail *Helix lucolum*, manipulation of 5-HT signaling has an influence on defensive conditioning (Andrianov et al., 2015), as well as memory extinction (Balaban et al., 2016). In a taste-aversive learning in *Lymnaea*, administration of a 5-HT receptor antagonist before memory tests leads the phenomenon of reversible amnesia (Nikitin and Solntseva, 2013; Nikitin et al., 2016a,b). In an operant conditioning of aerial respiration, 5-HT acts a main role in the enhancement of LTM with exposure of *Lymnaea* to a predator scent (Il-Han et al., 2010).

The key neurons of CTA learning are: (1) a pair of the cerebral giant cells (CGCs), which are 5-HTergic; and (2) their follower interneurons and motor neurons (Yeoman et al., 1994; Hatakeyama and Ito, 1999; Kawai et al., 2011; Ito et al., 2012; Sunada et al., 2017a). From the viewpoint of a presynaptic neuron theory in learning and memory, the amount of 5-HT released is controlled by a cAMP-PKA-CREB cascade in the CGC (Nakamura et al., 1999; Hatakeyama et al., 2004, 2006; Sadamoto et al., 2004, 2010, 2011; Wagatsuma et al., 2005, 2006). Here, CREB is a cAMP-response element binding protein.

Kandel formalized a “presynaptic hypothesis of learning and memory” and found that 5-HT-induced a cAMP increase in the presynaptic sensory neurons leading to a transient enhancement of transmitter release at the synapse between the presynaptic sensory and postsynaptic motor neuron of the gill-withdrawal reflex in *Aplysia* (Kandel, 2001, 2012). PKA is activated by this cAMP, and then CREB 1 is phosphorylated by PKA, enhancing transmitter release for memory consolidation. Interestingly, the balance between the amount of a transcriptional activator (CREB1) and that of a transcriptional repressor (CREB2) was examined in the CGC of *Lymnaea*, and it was found that the expression of a repressor gene (i.e., CREB2) predominated (Wagatsuma et al., 2005).

5-HT is also involved in both feeding behavior and food satiety in *Lymnaea* (Kemenes and Benjamin, 1989; Kemenes et al., 1990; Yamanaka et al., 1999, 2000; Yeoman et al., 2008; Kawai et al., 2011; Dyakonova et al., 2015a,b; Yamagishi et al., 2015) most probably *via* the 5-HTergic CGCs (Yeoman et al., 2008; Kawai et al., 2011; Sunada et al., 2017a). An increase in 5-HT in the CNS of *Lymnaea* results in a decrease in feeding responses to sucrose (Aonuma et al., 2018a). Thus, application of 5-HT may mimic the change from a food-deprived state to a food satiation state, normally achieved by the ingestion of food (Yamagishi et al., 2015). Moreover, previous studies showed that the firing rate of the CGC was significantly lower in Day 5 snails and that the membrane potential was significantly hyperpolarized in Day 5 snails than in the fed control (Dyakonova et al., 2015a).

Dyakonova et al. (2015b) further examined another set of 5-HTergic neurons, the pedal A (PeA) cluster cells in *Lymnaea*. They found that isolated PeA cluster cells decreased their firing rate in response to glucose application, suggesting that the hemolymph glucose concentration may decrease the activity of these cells. The PeA cluster cells release 5-HT onto various locomotor organs of *Lymnaea* (Syed and Winlow, 1989). Previous studies using other gastropods suggested that strong and long-lasting excitation of the PeA cluster cells causes locomotor arousal (Kabotyanski et al., 1990; Kabotyanskii and Sakharov, 1991; Satterlie, 1995). This locomotor arousal is involved in food searching behavior in *Lymnaea* (D’yakonova and Sakharov, 1995). Together, 5-HT plays important roles in feeding behavior and food satiety, suggesting that it is strongly involved in CTA in *Lymnaea*.

**Figure 3:**
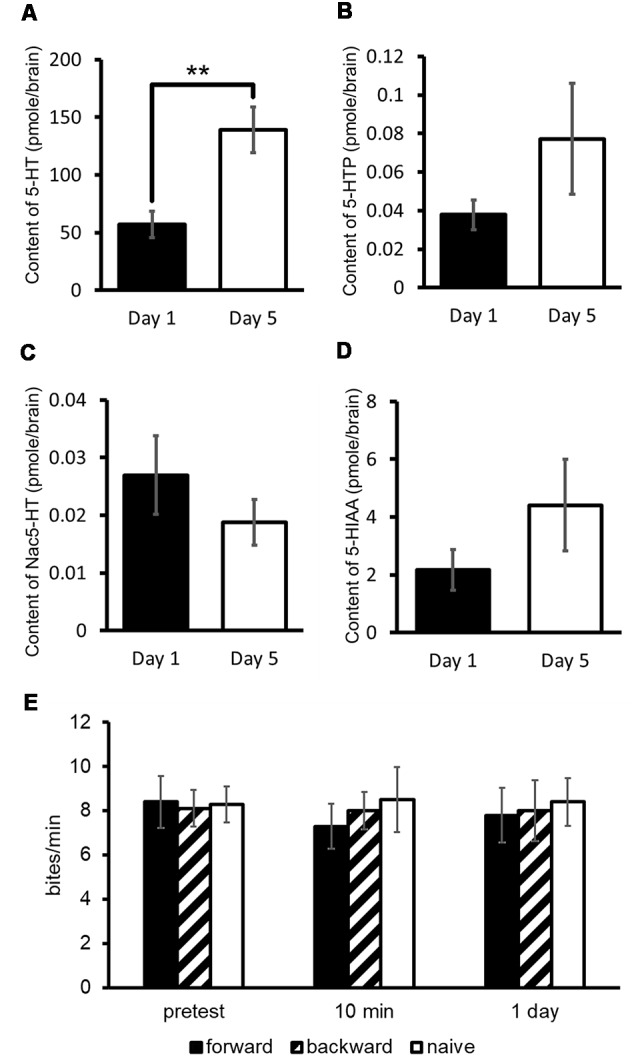
CNS 5-hydroxytryptamine (5-HT) content. **(A–D)** Monoamine contents of specific 5-HT metabolites were measured in the CNS of Day 1 and Day 5 snails. The number of CNSs was 10 each. The data are expressed as the mean ± SEM. **Indicates *P* < 0.01. **(E)** CTA memory formation of Day 1 snails was altered by immersion in a 300 μM 5-HT bath for 24 h. The number of snails used was 10 each. The feeding response of CTA-trained snails to the CS was not suppressed in comparison with the pretest. This figure was modified from Aonuma et al. (2018a). This article was published in *Neurobiol. Learn. Mem*., and thus the reuse of contents is permitted by the “author and user rights” of Elsevier.

Assuming that 5-HT plays a key role in CTA, we measured 5-HT and its precursor (5-hydroxytryptophan: 5-HTP) and catabolites (*N*-acetylserotonin: Nac-5-HT and 5-hydroxyindole acetoaldehyde: 5-HIAA) in the CNS of Day 1 and Day 5 food-deprived snails (Figure 3A; Aonuma et al., 2018a). The results obtained from the experiments of high-performance liquid chromatography with electrochemical detection (HPLC-ECD) showed that the 5-HT content in the CNS of Day 1 snails was significantly lower than that of Day 5 snails (Welch *t*-test, *n* = 10 each, *P* < 0.01; Figure 3A). However, the levels of 5-HTP, Nac-5-HT and 5-HIAA were not significantly different between Day 1 and Day 5 snails (Welch *t*-test, *n* = 10 each, *P* > 0.05; Figures 3B–D; Aonuma et al., 2018a).

We considered that Day 1 snails, which are the better learners, would show impaired CTA learning and memory when the 5-HT content was increased. To examine this, we immersed Day 1 snails in a 5-HT solution before CTA training (Aonuma et al., 2018a). In these snails, we did not observe CTA memory formation [One-way analysis of variance (ANOVA), *n* = 10 each, *P* > 0.05, Figure 3E]. The rescue of poor CTA memory in Day 5 snails will be discussed in the later section of MIP.

### Octopamine and CTA

OA plays a number of important roles in learning and memory in invertebrates (Giurfa, 2006; Farooqui, 2007; Kim et al., 2013). For example, OA mediates the appetitive (reward) reinforcement for olfactory conditioning in insects (Mizunami and Matsumoto, 2010). Blockade of OAergic signaling impedes appetitive learning, and optogenetic activation of OAergic neurons induces appetitive learning in *Drosophila* larvae (Schroll et al., 2006).

There are only a few studies of the possible role played by OA in molluskan learning and memory formation. Some studies of the role played by OA in the mediation of *Lymnaea* feeding behavior have been published (Elliott and Vehovszky, 2000a; Vehovszky and Elliott, 2000, 2001, 2002; Vehovszky et al., 2000, 2004a,b, 2005; Pitt et al., 2004). These findings were expanded to the results that OA plays a role in the LTM formation in a form of aversive food conditioning in *Lymnaea* (Kemenes et al., 2011). OA is also involved in the mediation of locomotion of *Lymnaea* (Miyamae et al., 2010) and may be associated with locomotor arousal, as described in the 5-HT section.

**Figure 4:**
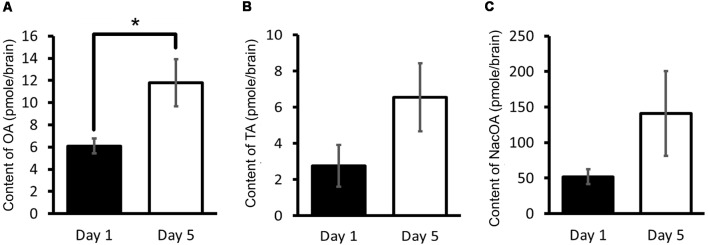
CNS octopamine (OA) content. **(A–C)** Monoamine contents of specific OA metabolites were measured in the CNS of Day 1 and Day 5 snails. The number of CNSs was 10 each. The data are expressed as the mean ± SEM. *Indicates *P* < 0.05. This figure was modified from Aonuma et al. (2017). This article was published in *Neurobiol. Learn. Mem*., and thus the reuse of contents is permitted by the “author and user rights” of Elsevier.

Using HPLC-ECD, OA, tyramine and *N*-acetyloctopamine (NacOA) in the CNS of Day 1 and Day 5 snails were measured (Aonuma et al., 2017). Tyramine is the precursor of OA, and NacOA is the catabolite of OA. The OA content of Day 1 snails was significantly lower (Welch *t*-test, *n* = 10 each, *P* < 0.05) than that in Day 5 snails (Figure 4A). Further, although there was not a significant difference in the tyramine content or the NacOA content between the CNS of Day 1 and Day 5 snails (Welch *t*-test, *n* = 10 each, *P* > 0.05, Figures 4B,C; Aonuma et al., 2017), a downward trend was found in Day 1 snails compared with Day 5 snails. We thus concluded that when the OA content is lower, the CTA memory score is better.

### Dopamine and CTA

Dopamine (DA) pathways act an essential role in reward systems in both vertebrates and invertebrates (especially insects; Baik, 2013; Ranaldi, 2014; Mizunami et al., 2015; Hu, 2016). In *Lymnaea*, as well as other mollusks, some studies of DAergic pathways have been performed by the pharmacological and immunohistochemical methods (Gospe, 1983; Elekes et al., 1991; Barnes et al., 1994; Kemenes, 1997; Vehovszky et al., 2007). DA has been shown to play an important role in reward classical and operant conditioning in *Aplysia* (Baxter and Byrne, 2006), and in the LTM consolidation of reward classical conditioning in *Lymnaea* (Kemenes et al., 2011).

DA is also involved in the feeding control of *Lymnaea*. For example, DA is found in the buccal ganglion neurons, and its application enhances the feeding response (Elliott and Vehovszky, 2000b). DA receptors are observed in the CGCs (Hernádi et al., 2012). The application of DA onto the CGCs increases their electrophysiological activity (Hernádi et al., 2004). Together, these data suggest that the DAergic input changes the activity of CGC and as a consequence, alters feeding behavior.

**Figure 5:**
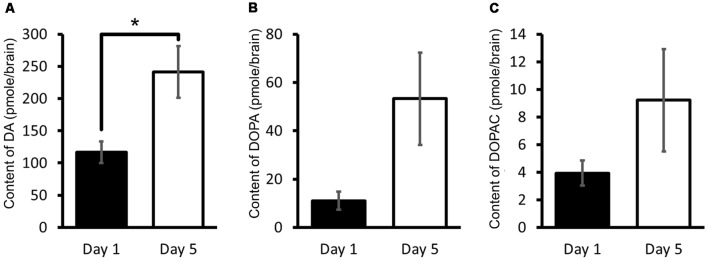
CNS dopamine (DA) content. **(A–C)** Monoamine contents of specific DA metabolites were measured in the CNS of Day 1 and Day 5 snails. The number of CNSs was 10 each. The data are expressed as the mean ± SEM. *Indicates *P* < 0.05. This figure was modified from Aonuma et al. (2016). This article was published in *Biol. Open*, and thus the reuse of contents is permitted by the CC BY 4.0 license.

We have therefore in *Lymnaea* measured the CNS DA content and its related catabolites using HPLC-ECD (Aonuma et al., 2016). It was found that the DA content of Day 1 snails was significantly lower (Welch *t*-test, *n* = 10 each, *P* < 0.05) than that found in Day 5 snails (Figure 5A). It was suggested therefore that a high CNS DA content leads to poor CTA memory formation.

In addition, the 3,4-dihydroxyphenylalanine (L-DOPA) and 3,4-dihydroxyphenylacetic acid (DOPAC) contents were also measured in the CNS. L-DOPA is the precursor molecule of DA and is converted to DA by L-aromatic amino acid decarboxylase (Cooper et al., 2003). DOPAC is a catabolite of DA, and DA is converted to DOPAC by MAO (Figure 2B). Even though DA is also converted to homovanillic acid (HVA) by catechol-*O*-methyltransferase (COMT) and MAO (Cooper et al., 2003), only DOPAC is noted here. The contents of L-DOPA and DOPAC were measured (Figures 5B,C; Aonuma et al., 2016). No significant difference was observed in the L-DOPA content or the DOPAC content between the CNS of Day 1 and Day 5 snails (Welch *t*-test, *n* = 10 each, *P* > 0.05). These data show that the score of CTA memory can be negatively correlated with the CNS DA content.

### Different Learning Ability in Different Populations for CTA

*Lymnaea stagnais* used above is the inbreed Dutch strain. The Dutch strain was collected in the 1950s (see van der Steen et al., 1969), and maintained at the Vrije Universiteit Amsterdam since then. *Lymnaea stagnalis* is a holarctic organism distributed across Northern Europe and North America (Mozley, 1939) and can be easily collected in ponds for study. Previous studies focusing on a mitochondrial ribosomal RNA revealed genetically distinct populations of *Lymnaea stagnalis* (Remigio and Blair, 1997). There was detectable genetic variability among *Lymnaea* in different ponds/bays of lakes within 20 km of each other in Finland (Puurtinen et al., 2004a,b, 2007). The genetic variation in copper tolerance was also found in *Lymnaea* (Côte et al., 2015). To explain the observed genetic divergence, researchers suggested random genetic drift (Remigio, 2002). A study of different populations of *Lymnaea stagnalis* collected in Belgium, Netherlands and Germany came to a similar conclusion (Bouétard et al., 2014). *Lymnaea* can differentiate into different strains/populations, although the mechanism(s) has not yet been elucidated (Dodd et al., 2018).

It is known that there are different learning abilities among strains of *Lymnaea*, when aerial respiratory behavior is operantly conditioned (Orr et al., 2009; Shymansky et al., 2017). Thus, it was of interest to determine if there were also similar strain-dependent differences in CTA learning and memory formation. Different strains were therefore used: (1) the inbreed Dutch strain; (2) the Canadian TC1 strain. A third population was also used; and (3) the filial 1 (F_1_) generation between the Dutch and Canadian TC1 snails. These three populations were kept in a laboratory (i.e., not freshly collected). The Canadian TC1 strain was collected in 2010 from a pond near the Trans-Canada Highway, AB, Canada (Braun et al., 2012). The Dutch and Canadian TC1 strains have been bred in Canada and Japan for many generations. The F_1_ cross population was produced in Japan (Sunada et al., 2017c).

**Figure 6:**
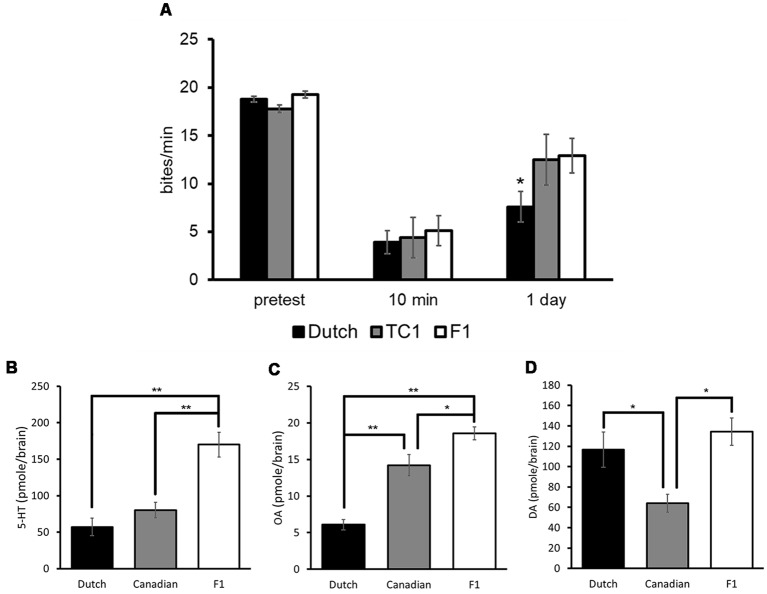
Comparison of three different *Lymnaea* populations. Black bars indicate the Dutch snails; gray bars indicate the Canadian TC1 snails; white bars indicate the F_1_ cross snails. The data are expressed as the mean ± SEM. **(A)** The feeding response was examined in these three populations. The number of snails was at least 10 each. There were no significant differences in the feeding response to the CS at the pretest (*P* > 0.05). At the 10 min post-test, the feeding responses to the CS were significantly suppressed in all three cohorts (*P* < 0.01), and there were no significant differences among the three cohorts (*P* > 0.05). At the 24 h post-test, a multiple comparison showed that *P < 0.05 between the Dutch and F_1_ cross snails; *P < 0.05 between the Dutch and Canadian TC1; and *P* > 0.05 between the Canadian TC1 and F_1_ cross snails. Control data (not shown) showed that CTA did not occur with backward-conditioned and naive control snails. **(B–D)** 5-HT, OA and DA contents in the CNS of three different populations respectively are shown. The number of CNSs collected from the Dutch strain and the Canadian TC1 strain snails was 10 each; and the number of CNSs collected from the F_1_ cross snails was six. *P < 0.05, **P < 0.01. This figure was modified from Sunada et al. (2017c) and Aonuma et al. (2018b). These articles were published in *Front. Behav. Neurosci*. and *Biophys. Physicobiol*., respectively, and thus the reuse of contents is permitted by the CC BY 4.0 license.

Each of the populations, which were food-deprived for 1 day, responded to sucrose similarly at the pretest, and the feeding responses to the CS in all three populations were significantly decreased at the 10 min post-test in comparison with those of the pretest (two-way ANOVA followed by a *post hoc* Holm test, *n* = at least 10 each, *P* < 0.01, Figure 6A; Sunada et al., 2017c). That is, all the three populations showed an aversive learning following CTA conditioning. When LTM was tested 24 h after CTA training (i.e., the 24 h post-test), memory was observed in all the three populations (two-way ANOVA followed by a *post hoc* Holm test, vs. the pretest, *P* < 0.01, Figure 6A). However, the feeding response was significantly less in the Dutch snails in the 24 h post-test than those of the Canadian TC1 and the F_1_ cross snails (two-way ANOVA followed by a *post hoc* Holm test, *P* < 0.05, Figure 6A). We thus found that the Dutch snails formed better CTA-LTM than the other two populations, indicating that the Dutch snails exhibited a smarter behavioral phenotype. On the other hand, in the backward conditioning and the naive control procedures (i.e., control behavioral experiments), no significant differences were observed in the feeding responses to the sucrose CS among the three populations at either the pretest, the 10 min post-test or the 24 h post-test (Sunada et al., 2017c).

We then measured the contents of 5-HT, OA and DA in the CNS of *Lymnaea* using HPLC-ECD (Aonuma et al., 2018b). The data were obtained from Day 1 snails. The 5-HT content in the Dutch and Canadian TC1 snails was significantly lower (one-way ANOVA followed by a *post hoc* Holm test, *n* = 10, *P* < 0.01) than in the F_1_ cross populations (Figure 6B). Further, the OA content in the Dutch snails was significantly lower (one-way ANOVA followed by a *post hoc* Holm test, *n* = 10, *P* < 0.01) than in either the Canadian TC1 or the F_1_ cross populations (Figure 6C). In contrast, the DA content in the Canadian TC1 strain was found to be significantly less (one-way ANOVA followed by a *post hoc* Holm test, *n* = 10, *P* < 0.05) than in either the Dutch strain or the F_1_ cross snails (Figure 6D; Aonuma et al., 2018b). It is unclear presently what this difference in DA content means to this Canadian TC1 strain.

It is important to note that DA is a key molecule in aerial respiration and in the operant conditioning procedure (Scheibenstock et al., 2002). A DAergic neuron, right pedal dorsal 1 (RPeD1), is thought to be a necessary neuron for LTM formation in this aerial respiratory operant conditioning. Further, the Canadian TC1 snails form LTM faster and better following the operant conditioning (Braun et al., 2012), and they have a significantly lower DA content than the Dutch strain (Figure 6D). Thus, when the two distinct conditioning procedures (i.e., CTA and an aerial respiratory operant conditioning) are examined, the lower monoamine contents correlate with the better memory formation in *Lymnaea*.

### MIP and CTA

In 2006, Azami et al. (2006) constructed a DNA chip for *Lymnaea* and, using it, they identified which genes were upregulated or downregulated by CTA training. Some of the upregulated genes were the MIPs I and II. MIPs were found as the first insulin-like peptide in invertebrates (Smit et al., 1988). The role of insulin-like peptides in gastropods has been shown to control growth as well as hemolymph glucose concentration (Smit et al., 1988; Horn et al., 1998). Insulin receptors, including MIP receptor (Roovers et al., 1995), are homologous across phyla, even in human (Jonas et al., 1996), and ligand-binding sites are especially conserved (see CAA59353 for *Lymnaea*, AAA59174 for human, and AAA39318 for mouse). Therefore, we assumed that: (1) mammal insulin would work instead of MIPs; and that (2) when we use an antibody against the extracellular domain of mammal insulin receptor, it would act as an antagonist for MIP receptor (Murakami et al., 2013a). We used mouse monoclonal antibody to insulin receptor alpha subunit (ab982, Abcam, Cambridge, UK), which recognizes the extracellular domain of human insulin receptor, acts as an antagonist that blocks the binding between insulin and the insulin receptor (Taylor et al., 1987).

When the partially purified MIPs, including MIPs I and II, obtained from the CNS or bovine insulin were applied to the isolated CNS, a long-term synaptic enhancement was observed between the CGC and the follower motor neuron involved in feeding (Hatakeyama et al., 2013; Murakami et al., 2013a,b). This synaptic enhancement was suppressed by an application of the insulin receptor antibody, described above, to the CNS. Further, an injection of the insulin receptor antibody into the snail abdomen before CTA training blocked the memory consolidation process (i.e., LTM was not consolidated), even though it did not block the memory 10 min after CTA training (Murakami et al., 2013a).

**Figure 7:**
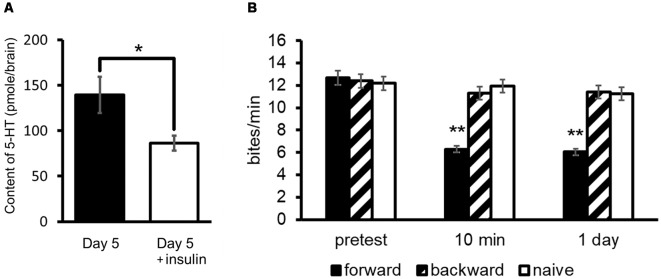
Rescue of CTA memory impairment of Day 5 snails by an injection of insulin. **(A)** 5-HT content in the CNS of Day 5 snails was decreased by an injection of insulin. The number of CNSs was 10 each. The data are expressed as the mean ± SEM. *Indicates *P* < 0.05. **(B)** Memory formation occurred in Day 5 snails injected with insulin 60 min before CTA training. Black bars indicate CTA-trained snails; hatched bars indicate backward-conditioned snails; and white bars indicate naive control snails. The number of snails was 20 each. The time indicated on the *x*-axis represents the time of the post-test session that was performed following the training. **Indicates *P* < 0.01. This figure was modified from Mita et al. (2014b) and Aonuma et al. (2018a). Both of these articles were published in *Neurobiol. Learn. Mem*., and thus the reuse of contents is permitted by the “author and user rights” of Elsevier.

As described previously, Day 5 snails show a poorer memory score compared with Day 1 snails (Sugai et al., 2007; Mita et al., 2014a,b). As shown in Figure 3A, the 5-HT level of Day 5 snails is higher than that of Day 1 snails (Aonuma et al., 2018a). We injected insulin (in this case we used bovine insulin but not MIPs) into Day 5 snails before CTA training, and found that 5-HT content decreased in these snails (Welch *t*-test, *n* = 10, *P* < 0.05, Figure 7A; Aonuma et al., 2018a). Further, we found that the LTM deficiency (i.e., poor LTM formation) was rescued (two-way ANOVA followed by a *post hoc* Holm test, *n* = 20 each, *P* < 0.01, Figure 7B; Mita et al., 2014b). We thus conclude that this improvement of memory score by insulin injection in Day 5 snails could be a result of decreased 5-HT level in the CNS.

## Discussion

Our results showed that there is a negative correlation between the CNS monoamine contents and the CTA memory scores in *Lymnaea*. Severely food-deprived Day 5 snails exhibit poor CTA memory, but when they are injected with insulin, their 5-HT CNS content decreases so that these snails now exhibit a higher memory score. Together, these data raise an important question. Are monoamines unnecessary for plasticity in the neural network controlling feeding behavior in *Lymnaea*? It is known that the key neuron in CTA is the 5-HTergic CGC (Yeoman et al., 1994; Hatakeyama and Ito, 1999; Kawai et al., 2011). If we look at *Lymnaea*’s development, the earliest embryos can acquire CTA at stage 29, and it is at this stage that CGC immunoreactivity of 5-HT is first observed (Yamanaka et al., 1999, 2000). Thus, we believe that it is correct to say that 5-HT is needed for CTA. Additionally, because CTA is a suppression of feeding and because 5-HT depletion impairs feeding behavior in *Lymnaea* (Kemenes et al., 1990; Croll et al., 1997), the phenomenon of a decrease in 5-HT concentration observed in Day 1 snails is consistent with why there is better CTA formation. However, that conclusion poses a problem. The problem is why there is a negative correlation observed between the monoamine contents and the CTA memory scores. We think that we can solve this problem with the following arguments.

First, we measured the total CNS monoamine contents not the contents at a specific synaptic site. Thus, whereas lower overall monoamine contents are indicative of higher scores of CTA memory, we do not know how monoamine contents at a specific synapse correlate with the scores of CTA memory. At present, we cannot measure synaptic monoamine contents. In other words, we should consider that the relative change ratio of synaptic monoamine concentration is important. The relatively large change may occur in the situation when the total monoamine contents are low in the CNS. Further, the time constant of monoamines (i.e., time from production and decay of a target molecule) in a small region (i.e., a synaptic cleft) also seems important in learning and memory. The time constant of monoamines is larger than those of other transmitters (Glusman and Kravitz, 1982; Pasztor and Golas, 1993; Kobayashi et al., 2000a,b).

Second, we showed that Day 5 snails (i.e., a longer period of food deprivation) have a poorer CTA memory (Sugai et al., 2007). We know that the memory score is dependent on the level of stress the animal is encountering. This is explained by the Yerkes-Dotson/Hebb inverted U curve (Yerkes and Dodson, 1908; Hebb, 1955; Ito et al., 2015a). In this view, Day 1 snails are at optimal stress levels, whereas Day 5 snails are at a level of stress not conducive to learning and memory formation or the ability to recall a formed memory. We hypothesized that severely food-deprived (Day 5) snails might in fact learn CTA and form LTM, but that the severe food deprivation obstructs the ability to recall (Ito et al., 2015b).

Third, we hypothesize that food deprivation results in a specific behavioral state, and thus constitutes a specific context around which learning and memory are anchored (Palmer and Kristan, 2011; D’yakonova, 2014). Thus, the context was the behavioral state associated with food deprivation (Ito et al., 2015b). To test this hypothesis, we performed the following experiment. Day 5 snails were trained with the CTA-training procedure. Following training, the snails were given access to food for 7 days right. When CTA memory was tested at this time point, it seemed not to be present. However, if we then food-deprived these snails for 1 day and then tested for memory, CTA memory was present. That is, snails had to be in the similar context in which the memory was formed to activate the memory. Additionally, we believe that the prolonged food-deprived state leads snails to basically ignore the memory that sucrose (the CS) after CTA training reminds KCl or electrical shock (the US). If the snail is severely hungry, it is better to eat and not be worried about the aversive stimulation. That is, snails that are severely food-deprived (i.e., Day 5) must eat something to survive, even if something has become a reliable predictor of an aversive event. This phenomenon is considered to be “a consequence of conflict resolution” (Ito et al., 2015b, 2017). The fact that the memory phenotype was not exhibited in Day 5 snails has a biological meaning that has to do with the “necessity knows no law” concept (Ito et al., 2015b, 2017).

Our present review hopefully deepens the knowledge about the relation between “learning and memory” and “monoamines and insulin,” and offers the future questions. Particularly, the crosstalk between the 5-HT pathway and the insulin pathway in the CNS is interesting.

## Author Contributions

EI designed the contents of this article. YT, HA, AO, TW and DH collected and analyzed the original data. MS, KL and EI interpreted the data and wrote the manuscript. All authors read and approved the final manuscript.

## Conflict of Interest Statement

The authors declare that the research was conducted in the absence of any commercial or financial relationships that could be construed as a potential conflict of interest.
